# The Prognosis in a Case of Cerebral Hæmorrhage

**Published:** 1906-05-19

**Authors:** H. Campbell Thomson

**Affiliations:** Assistant Physician to the Middlesex Hospital, Physician to the Bolingbroke Hospital, and to the Hospital for Epilepsy and Paralysis, Maida Vale.


### Hospital Clinics.

### THE PROGNOSIS IN A CASE OF CEREBRAL HÆMORRHAGE.

By H. Campbell Thomson, M.D., F.R.C.P., Assistant Physician to the Middlesex Hospital, Physician to the Bolingbroke Hospital, and to the Hospital for Epilepsy and Paralysis, Maida Vale.

One of the most serious misfortunes that can befall a man still in the active period of life is to break a cerebral artery, for while a fatal termination may be averted, it is very unlikely that voluntary power and mental ability will ever regain their former vigour. When the anxiety concerning immediate danger to life has passed away, which, in favourable circumstances, it will do within a few days, every one interested in the matter will naturally wish to know how much return of movement is likely to take place, and though it is usually impossible in the early days to predict the extent of recovery with any degree of certainty, some general statement of a reassuring kind can often be based on a knowledge of the usual course such cases take, while any special points about the particular patient under consideration are kept in mind.

Loss of voluntary power arises from the inability of impulses to pass along damaged nerve-fibres, the damage being due partly to pressure of the blood-clot and partly to actual rupture of fibres, and it is on the relative proportion between pressure and destruction that the degree of recovery ultimately depends, for, while the fibres subjected to pressure are likely to recover some of their functions as the clot shrinks, those whose continuity has been broken are for the most part permanently destroyed.

Time only can show how much injury is permanent, but unless there are special indications to the contrary, it is reasonable to infer that the function of a certain number of fibres will return and that a corresponding amount of voluntary power will be regained. It can further be said that, in the event of such return, the movements of the face will be for all practical purposes restored; that those of the leg will improve before, and to a greater extent than those of the arm; and if all goes well there is good reason to hope that the patient will eventually be able to walk. About the use of the arm nothing very definite had better be said, for while one hopes for the best, the fact remains that in the large majority of cases usefulness is greatly impaired if not completely lost, at any rate as far as the hand is concerned.

In order to understand the origin of motor symptoms arising from cerebral hæmorrhage, it is necessary to enter into the mechanism of their production in some detail, and for this purpose movements may be divided into four distinct classes.

1. Movements which have come to be entirely under the control of the will such as the highly specialised ones of the legs, arms, and fingers.

2. Movements which are incompletely under control of the will, such, for instance, as those concerned in moving the eyebrows and closing the eyes. The average man has not educated these movements sufficiently to be able to close one eye, or wrinkle one brow, without setting up corresponding movement of the other side. These movements are usually spoken of as bilateral, since they are performed together, and the connection between the two sides probably takes place at the nerve nuclei.

3. Movements produced by emotions. These are widespread and (if the emotion be strong enough) are only slightly controllable.

4. Simple reflex movements.

Now the motor impulses which give rise to voluntary movements can only pass along a very limited set of fibres (known as the motor tract) and unfortunately the artery which most often breaks—the lenticulo striate artery—is in close proximity to that tract and, therefore, a hæmorrhage in this region is almost sure to interrupt the impulses, and cause loss of voluntary power on the opposite side of the body. The impulses find the path blocked and have no alternative route, and so we get paralysis of arm and leg and lower part of the face, the upper part of the face escaping or being only slightly weakened because the movements there belong to the bilateral class described in the next paragraph.

The movements which are bilateral usually escape or are only very temporarily modified, and so the patient suffering from hæmorrhage in the region of the internal capsule can still close the eyes, wrinkle the brow, and frown, and can also use other bilateral movements, as, for instance, those connected with respiration, and fortunate it is that this is the case, otherwise cerebral hæmorrhage would be even more dangerous to life than it is at present.

The bilateral character of these movements is due to the existence of communication between the nuclei of the nervous paths, and it is to the preservation of these communications that their immunity is due, for when the impulses are blocked in the neighbourhood of the internal capsule on one side of the brain, some can still reach the muscles from the other side through the nuclear communication.

Impulses which give rise to movements expressing emotions such as laughing and crying, travel by more diverse paths than those causing strictly voluntary movements, and so they also escape when only the motor tract is involved.

This can be well demonstrated by noting the inability of a patient to make any voluntary lateral movement of his mouth, and the freedom by which the same movement is made when laughing.

The reflex movements on the paralysed side become increased and at the same time the limbs tend to become stiff and contracted.

With this knowledge the possibilities of progress can be appreciated and some definite idea can soon be obtained as to the course things are likely to take. The onset of permanent rigidity with contracture is the danger that threatens during the stage of recovery, for once these are firmly established, there is little hope of restoring the limb to anything like its former pliability. It is therefore important to try to counteract the tendency to rigidity from the very beginning by carefully preventing the limbs, especially the arm and hands, from assuming bad positions, and keeping them supple by massage, for in rigidity and contracture the flexors and adductors overcome the extensors and abductors and the arm gradually tends to assume the characteristic position of being drawn close to the side and bent at all the joints.

As soon as there is any sign of returning movement the patient should encourage it as much as possible, for the movement shows that the pathway is clearing and the passage of impulses probably helps to keep up the nutrition of fibres, since unexercised organs always tend to atrophy. The sooner movements begin to return, the more hope there is of regaining a useful limb, but moderate delay does not necessarily mean there will be no return, for occasionally movements begin to come back after quite a long time.

Cessation of improvement is a bad sign; it is suggestive that the furthest point has been reached, but when this appears to be taking place, encouragement and suitable massage, with perhaps electrical treatment, are often beneficial, for the arrested improvement may be partially due to stiffness of joints and muscles that can be dispelled by treatment. Warm baths have recently been recommended as an aid to diminishing stiffness and promoting restoration of power, and considering the ease with which they can be obtained, seem worthy of trial.

The knowledge that full power will never return is apt to discourage continuance of treatment, for when it seems obvious that the patient will always be more or less of a cripple for the rest of his life, it may scarcely seem worth while to do much. In these circumstances it must be remembered that a slight amount of improvement may mean all the difference to the comfort of the patient. He will soon cease to be ambitious, but he will, at the same time, still be very thankful for a range of movement which to the healthy seems almost a negligible quantity.

Lastly, the frequency of cerebral hæmorrhage and consequent hemiplegia should serve to remind us that, as Professor Clifford Allbutt has shown, many of them might have been prevented had their blood-pressure been systematically attended to, a fact which opens up an extensive field for preventive medicine in the future, for when symptoms are discovered the cause is often beyond remedy.

